# The Complete Mitochondrial Genome of Bean Goose (*Anser fabalis*) and Implications for Anseriformes Taxonomy

**DOI:** 10.1371/journal.pone.0063334

**Published:** 2013-05-23

**Authors:** Gang Liu, Lizhi Zhou, Lili Zhang, Zijun Luo, Wenbin Xu

**Affiliations:** 1 School of Resources and Environmental Engineering, Anhui University, Hefei, P. R. China; 2 Anhui Biodiversity Information Center, Anhui University, Hefei, P. R. China; 3 The Shengjin Lake National Nature Reserve of Anhui Province, Dongzhi, P. R. China; University of Strasbourg, France

## Abstract

Mitochondrial DNA plays an important role in living organisms, and has been used as a powerful molecular marker in a variety of evolutionary studies. In this study, we determined the complete mtDNA of Bean goose (*Anser fabalis*), which is 16,688 bp long and contains 13 protein-coding genes, 2 rRNAs, 22 tRNAs and a control region. The arrangement is similar to that of typical Anseriform species. All protein-coding genes, except for Cyt b, ND5, COI, and COII, start with an ATG codon. The ATG start codon is also generally observed in the 12 other Anseriform species, including 2 *Anser* species, with sequenced mitochondrial genomes. TAA is the most frequent stop codon, one of three–TAA, TAG, and T- –commonly observed in Anseriformes. All tRNAs could be folded into canonical cloverleaf secondary structures except for tRNA^Ser^(AGY) and tRNA^Leu^(CUN), which are missing the dihydrouridine (DHU) arm. The control region of Bean goose mtDNA, with some conserved sequence boxes, such as F, E, D, and C, identified in its central domain. Phylogenetic analysis of complete mtDNA data for 13 Anseriform species supports the classification of them into four major branches: Anatinae, Anserinae, Dendrocygninae and Anseranatidae. Phylogenetic analyses were also conducted on 36 Anseriform birds using combined Cyt b, ND2, and COI sequences. The results clearly support the genus *Somateria* as an independent lineage classified in its own tribe, the Somaterini. Recovered topologies from both complete mtDNA and combined DNA sequences strongly indicate that Dendrocygninae is an independent subfamily within the family Anatidae and Anseranatidae represents an independent family. Based on the results of this study, we conclude that combining ND2, Cyt b, and COI sequence data is a workable solution at present for resolving phylogenetic relationships among Anseriform species in the absence of sufficient complete mtDNA data.

## Introduction

Animal mitochondrial DNA (mtDNA), typically a small (15–20 kb) double-stranded maternally-inherited circular genome, plays an important role in processes associated with metabolism, programmed cell death, illness, and aging [1]. It possesses a remarkably conserved set of 37 genes: 13 protein-coding genes (ND1–6 and ND4L [for NADH dehydrogenase subunits 1–6 and 4L], ATP6 and ATP8 [for ATPase subunits 6 and 8], COI–III [for cytochrome oxidase subunit I–III], and Cyt b [for cytochrome b]), two ribosomal RNA genes (12S rRNA and 16S rRNA), and 22 tRNA genes. In addition, it contains at least one variable length sequence known as the control region (D-loop) [1], [2], [3]. Compared with nuclear genes, mtDNA is conservative in gene content, abundant in cells, and intronless, but contains much important phylogenetic information [1], [4], [5], [6]. Because of these advantages, mtDNA is valuable for studies of genetic structure, biological identification, taxonomy, and phylogeny [7], [8], [9]. Some mtDNA regions, such as Cyt b, ND2, COI, and the control region, are of special interest, having been widely used to resolve taxonomy of controversial organismal groups [10], [11], [12]. Because they undergo moderate rates of evolution, the protein-coding genes Cyt b, ND2, and COI have been particularly useful [13], [14], [15]. In recent years, combinations of Cyt b, ND2, and COI gene sequences have been applied to phylogenetic problems at a variety of levels, ranging from related species to genera and families; they have been especially valuable for clarifying phylogenetic relationships within some controversial animal groups, especially that of some birds [10], [14], [15], [16], [17]. The mitochondrial control region, which contains initiation sites for transcription and replication, is the most variable mtDNA segment and has the fastest evolutionary rate. Because some conserved domains in the control region exhibit rather homogeneous evolutionary rates, this region can be used to determine intra- and interspecific relationships [1], [2], [3], [18].

In recent years, as a result of revolutionary advances in molecular techniques associated with PCR, sequencing, and data analysis, increasing attention has been focused on the complete mitochondrial genome [19], [20]. The complete genome, or a subset thereof, has been widely used as an informative molecular marker in a variety of evolutionary studies, including those involving phylogenetics and population genetics [1], [21]. Over the past 10 years, comparative analysis of mtDNA sequences and gene order has been employed as a powerful tool for resolving ancient phylogenetic relationships and elucidating population genetic structure [3,22.23]. Because the quantity of mtDNA sequence data used in studies of phylogenetic relationships at species and population levels is continually increasing [22], the mitochondrial genome has become a highly useful molecular marker for the reconstruction of phylogenetic relationships at different animal taxonomic levels. Analyses of complete mitochondrial genomes not only provide sequence information for phylogenetic studies, but also further elucidation of its structure and function [21], [24], [25].

Anseriformes is a large and complex group comprising approximately 150 species and exhibiting very high worldwide diversity [10], [26]. Although one of the best-studied groups of birds, taxonomic statuses and phylogenetic placements within the order remain in dispute [10], [16], [17], [27], [28], [29]. Various morphological characteristics and molecular data derived from nuclear and mitochondrial genes have been employed to infer phylogenetic relationships within Anseriformes. Many authors divide the order into three general families: Anhimidae (screamers), Anatidae (ducks, geese, swans), and Anseranatidae (magpie geese) [16], [30]. Anatidae, the largest and most important group in Anseriformes, is traditionally divided into subfamilies Anatinae and Anserinae [10], [16]. Anatinae consists of tribes Mergini, Anatini, Tadornini, and Aythyini, while Anserinae includes geese, swans, and *Dendrocygna* [10]. Taxonomic debates primarily focus on: (i) classification status of Anseranatidae, (ii) subfamilies of Anatidae, and (iii) phylogenetic relationships among these subfamilies. A major source of conflict is centered around whether Anseranatidae should be considered as an independent family, or rather as a subfamily of the family Anatidae [10], [16], [17], [27], [28]. These debates are still ongoing, but mitochondrial sequences are beginning to shed light on the phylogeny of Anseriformes [11], [29], [31], [32], [33]. Recent evidence has indicated that complete mtDNA may be able to resolve controversial relationships among Anseriform birds, and a few studies using this approach have been reported [11], [32], [33]. Complete mtDNA sequences of Anseriform birds are beneficial for correctly solving classification status problems. As the quantity of Anseriform mtDNA sequence data is increased, phylogenetic relationships should be gradually clarified. The main limitation to taxonomic resolution is thus the availability of mitochondrial genome data. There are only 12 Anseriform species for which complete mtDNA has been sequenced: *Anas platyrhynchos* (NC_009684), *Anas formosa* (NC_015482), Anser anser (NC_011196), *Anser albifrons* (NC_004539), *Anseranas semipalmata* (NC_005933), *Aythya americana* (NC_000877), *Branta canadensis* (NC_007011), *Cairina moschata* (NC_010965), *Cygnus columbianus* (NC_007691), *Cygnus atratus* (NC_012843), *Dendrocygna javanica* (NC_012844), and *Mergus squamatus* (NC_016723). Consequently, mtDNA genome sequences of additional Anseriform species should provide further insights into their diversity and evolution.

The Bean goose (*Anser fabalis*), a member of the family Anatidae (Anseriformes), is an important wetland indicator species locally distributed from Eurasia to North Africa. With the occurrence of rapid economic development during the last two decades, this population is suspected to be undergoing a continuous and rapid decline as a result of habitat loss, illegal hunting, and human disturbances [34]. Because systematic studies are essential for its population conservation, acquisition of the complete mtDNA sequence of this species would be of great value. In this study, we sequenced the Bean goose complete mtDNA genome and compared its gene structure with that of other tribal representatives to gain insights into mtDNA evolution. To contribute to an understanding of Anseriform evolutionary history, we also performed phylogenetic analyses based on complete mtDNA and combined Cyt b, ND2, and COI sequence data published in GenBank.

## Results

### Genome Organization and Gene Arrangement

**Figure 1:**
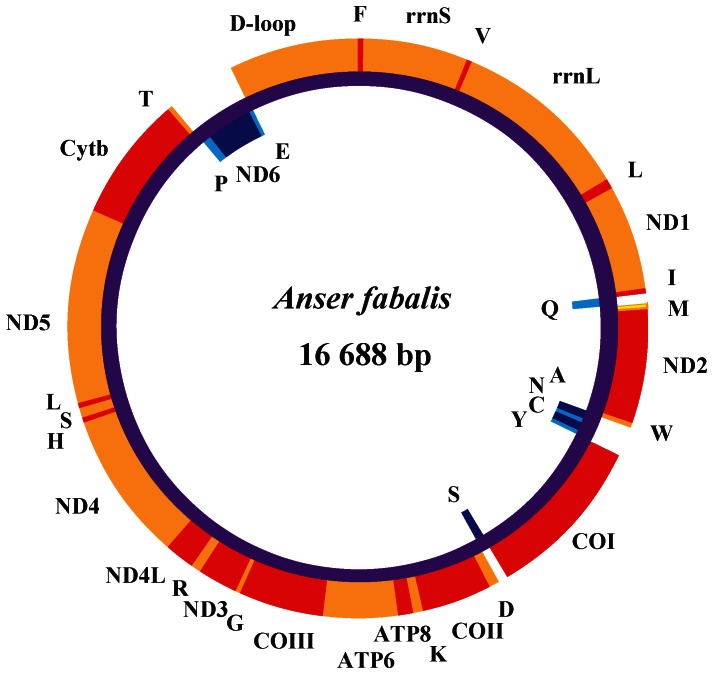
Mitochondrial genome of Bean goose. Genes encoded on the H-strand (clockwise orientation) are colored red or orange. Genes encoded on the L-strand (anti-clockwise orientation) are colored in dark or light blue. Abbreviations for genes are as follows: COI–III = cytochrome oxidase subunits, Cyt b = cytochrome b, ND1–6 = NADH dehydrogenase components, rrnL = 16S rRNA, and rrnS = 12S rRNA. tRNAs are denoted as one-letter symbols according to IUPAC-IUB single-letter amino acid codes.

The complete mtDNA sequence of Bean goose is 16,688 bp long and contains 13 protein-coding genes (ATP6, ATP8, COI–III, ND1–6, ND4L, and Cyt b), 2 rRNAs (12S rRNA and 16S rRNA), 22 tRNAs, and a putative control region (D-loop) (Fig. 1). The heavy DNA strand (H-strand) carries most of the genes: 12 protein-coding genes, two rRNAs, and 14 tRNAs. ND6 and eight tRNAs are located on the L-strand (Table 1).

**Table 1:** Organization of the mitochondrial genome of Bean goose.

Gene	Direction	Nucleotide no	Size	Spacer (+) or Overlap (−)	Start codon	Stop codon
tRNA^Phe^	F	1–67	67	0		
rRNA-Ssu	F	68–1051	983	10		
tRNA^Val^	F	1062–1122	61	0		
rRNA-Lus	F	1123–2793	1671	−74		
tRNA^Leu^	F	2718–2791	74	7		
ND1	F	2798–3760	963	−1	ATG	AGG
tRNA^Ile^	F	3759–3831	73	8		
tRNA^Gln^	R	3839–3909	71	−1		
tRNA^Met^	F	3909–3977	69	0		
ND2	F	3978–5016	1039	0	ATG	TAA
tRNA^Trp^	F	5017–5089	73	5		
tRNA^Ala^	R	5095–5163	69	2		
tRNA^Asn^	R	5165–5237	73	9		
tRNA^Cys^	R	5247–5306	60	−1		
tRNA^Tyr^	R	5306–5379	74	−2		
COI	F	5378–6928	1551	9	GTG	AGG
tRNA^Ser^(UCN)	R	6920–6992	73	2		
tRNA^Asp^	F	6995–7063	69	1		
COII	F	7065–7751	687	1	GTG	TAA
tRNA^Lys^	F	7753–7821	69	1		
ATP8	F	7823–7990	168	−8	ATG	TAA
ATP6	F	7981–8664	684	−1	ATG	TAA
COIII	F	8664–9448	785	0	ATG	T-
tRNA^Gly^	F	9449–9517	69	0		
ND3	F	9518–9868	351	1	ATG	TAA
tRNA^Arg^	F	9870–9940	71	0		
ND4L	F	9941–10237	297	−5	ATG	TAA
ND4	F	10231–11609	1378	0	ATG	TAA
tRNA^His^	F	11610–11678	69	1		
tRNA^Ser^ (AGY)	F	11680–11748	69	−4		
tRNA^Leu^(CUN)	F	11745–11815	71	0		
ND5	F	11816–13633	1818	7	GTG	AGA
Cytb	F	13641–14771	1131	2	ATG	TAA
tRNA^Thr^	F	14774–14849	76	0	ATG	TAA
tRNA^Pro^	R	14850–14918	69	0		
ND6	R	14929–15438	510	0		
tRNA^Glu^	R	15439–15506	68	0		
D-loop	F	15507–16688	1182			

### Protein-coding Genes

The total length of the 13 protein-coding genes is 11,296 bp, which represents 65.38% of the entire mitochondrial genome. The longest gene is ND5 (1,818 bp), located between tRNA^Leu^ (CUN) and Cyt b, and the shortest is ATP8 (168 bp), which is between tRNA^Lys^ and ATP6. All protein-coding genes start with an ATG codon, except for ND5, COI, and COII, which begin with GTG. TAA, found in 10 genes, is the most frequent stop codon, although ND5 ends with AGA, and ND1 and COI end with AGG.

### Ribosomal RNA, Transfer RNA, and Non-coding Regions

Bean goose mtDNA contains a small subunit (12S rRNA) and a large subunit (16S rRNA) of rRNA. They are located between tRNA^Phe^ and tRNA^Leu^, and are separated by tRNA^Val^. The 12S rRNA, located between nucleotide positions 68 and 1,051, is 983 bp long; the 16S rRNA is located between nucleotide positions 1,123 and 2,793, and has a length of 1,671 bp.

The mtDNA genome contains 22 tRNAs, ranging in size from 61 to 74 bp; the longest tRNA is tRNA^Leu^ (74 bp), and the shortest is tRNA^Val^ (61 bp). Except for tRNA^Ser^ (AGY) and tRNA^Leu^ (CUN), which lacks a dihydroxyuridine (DHU) arm, they could be folded into the typical cloverleaf structure.

The non-coding regions include a control region (D-loop) and a few intergenic spacers. The D-loop region is located between tRNA^Glu^ and tRNA^Phe^, and is 1,182 bp long. Intergenic spacers comprise 66 bp spread over 14 regions (Table 1). Areas of gene overlap occupy a total of 89 bp, with the longest region of overlap a 74-bp long sequence located between tRNA^Lus^ and tRNA^Val^ (Table 1).

### Phylogenetic Reconstructions

**Figure 2:**
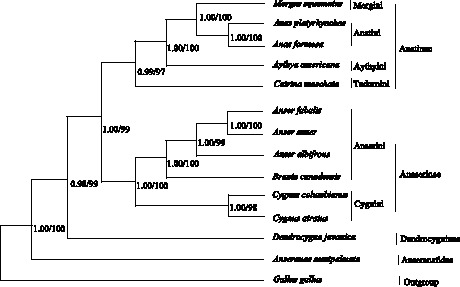
Phylogenetic relationships among 13 Anseriform species based on complete mitochondrial genome sequences. Numbers at each node are maximum likelihood bootstrap proportions (estimated from 100 pseudoreplicates) (left) and Bayesian posterior probabilities (right).

**Figure 3:**
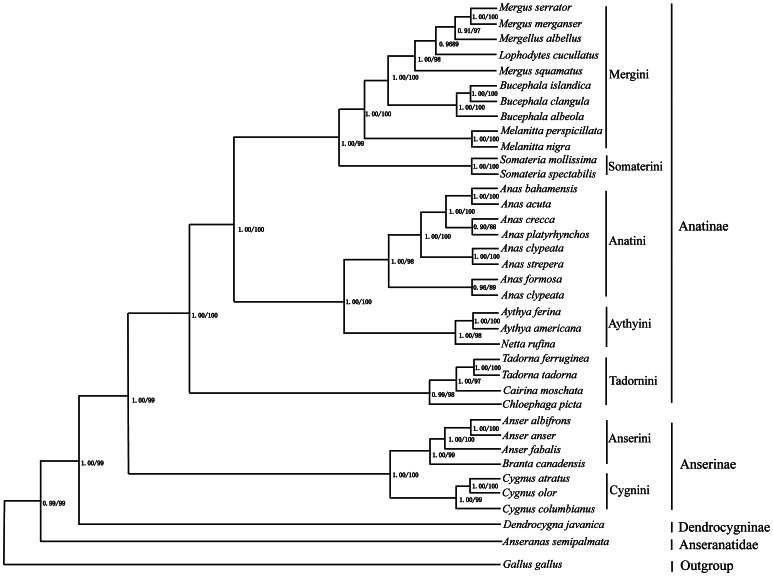
Phylogenetic relationships among 36 Anseriform species based on Cyt b, ND2, and COI genes. Numbers at each node indicate maximum likelihood bootstrap proportions (from 100 pseudoreplicates) (left) and Bayesian posterior probabilities (right).

Trees derived from phylogenetic analysis of complete mtDNA for 13 Anseriform species and of the three genes for 36 species have similar topologies (Figs. 2 and 3). The analyzed species are divided into four major clades: Anatinae makes up the first lineage, which is sister to the second group, Anaserinae; Dendrocygninae forms the third group and is sister to Anatinae and Anaserinae. The lineage consisting of these three groups in turn is sister to the fourth clade, Anseranatidae. The outgroup, *Gallus gallus*, is located at the base of the tree.

Trees generated from maximum likelihood (ML) and Bayesian inference (BI) analyses of complete mtDNA for 13 Anseriform species have highly similar topologies, with only slight differences in bootstrap support or posterior probability values (Fig. 2). Four major branches are represented: Anatinae, Anserinae, *Dendrocygna javanica*, and *Anseranas semipalmata*. The first lineage, subfamily Anatinae, includes species of tribes Mergini (*Mergus squamatus*), Anatini (*Anas formosa*, *Anas platyrhynchos*), Aythyini (*Aythya americana*), and Tadonini (*Cairina moschata*). The second lineage includes tribes Anserini (*Branta canadensis*, *Anser albifrons*, *Anser anser*, and *Anser fabalis*) and Cygnini (*Cygnus atratus* and *Cygnus columbianus*), forming the subfamily Anserinae. Anatinae and Anserinae are sister groups, which then group together with *Dendrocygna javanica*. *Anseranas semipalmata* is the sole member of the fourth group.

Topologies recovered from maximum likelihood (ML) and Bayesian inference (BI) analyses of the three genes from 36 Anseriform species are highly congruent, with strong bootstrap support observed for most nodes (Fig. 3). The 36 species are divided into four major lineages: Anatinae, Anserinae, *Dendrocygna javanica*, and *Anseranas semipalmata*. The first lineage, subfamily Anatinae, includes species of tribes Mergini, Somaterini, Anatini, Aythyini, and Tadonini. This lineage is a sister group to the second lineage, subfamily Anserinae, which comprises tribes Anserini and Cygnini. These two lineages form a clade that is in a sister group relationship with *Dendrocygna javanica*; this clade in turn is sister to *Anseranas semipalmata*.

## Discussion

### Mitochondrial Genome Annotation and Features

In many respects, the newly-sequenced mitochondrial genome is nearly identical to those reported for other Anseriform birds. The compact arrangement of Bean goose mtDNA is similar to typical avian mtDNA [34], [35], which has no introns, no long intergenic spacers, and only a few overlapping sequences. Its length is similar to that of other birds, and well within the range of avian mitochondrial genomes [5], [32], [36]. The overall base composition is: A, 30.06%; C, 31.84%; G, 15.36%; and T, 22.74%. A+T content (52.80%) is higher than C+G content (47.20%), similar to other Anseriformes (51.6–55.7%) [37], [38]. Compared with mitochondrial genomes of other Anseriform species, the A, T, and A+T compositions of Bean goose mtDNA are similar, and it shares with the other genomes a strong AT bias (Table 2). Guanine (G) is the rarest nucleotide; the percentage of the other three bases are roughly equal to each other (Table 2), similar to other vertebrate animals [13]. GC and AT skews are a measure of compositional asymmetry; in amniote mtDNA, GC-skew values are all negative (G<C), while AT-skew is positive (A>T) [39]. In Bean goose mtDNA, GC-skew (−0.357) and AT-skew (0.143) values are in accord with this principle.

**Table 2:** Nucleotide composition (%) of some Anseriform mitochondrial genomes.

Species	T (%)	C (%)	A (%)	G (%)	A+T (%)	G+C(%)	Total nucleotide
*Anser albifrons*	22.63	32.05	30.15	15.18	52.78	47.23	16,737
*Anser anser*	22.58	32.14	30.19	15.09	52.77	47.23	16,738
*Anser fabalis*	22.74	31.84	30.06	15.36	52.80	47.20	16,688
*Anas formosa*	22.51	32.44	29.52	15.53	52.03	47.97	16,594
*Anas platyrhynchos*	22.19	32.52	29.21	16.08	51.40	48.60	16,606
*Aythya americana*	22.24	32.75	29.39	15.62	51.60	48.40	16,616
*Anseranas semipalmata*	23.49	31.38	30.92	14.21	54.41	45.59	16,870
*Branta Canadensis*	22.60	32.07	30.18	15.15	52.78	47.22	16,760
*Cygnus columbianus*	22.79	31.89	30.10	15.22	52.89	47.11	16,728
*Cygnus atratus*	22.20	32.55	29.52	15.73	51.72	48.28	16,748
*Cairina moschata*	21.93	32.95	29.00	16.12	54.88	45.12	16,610
*Dendrocygna javanica*	23.67	30.44	30.44	15.45	54.11	45.89	16,753
*Mergus squamatus*	22.26	32.76	29.04	15.94	51.30	48.70	16,595

### Comparison of Three Anser Mitochondrial Genomes

Mitochondrial genomes sequenced from three *Anser* species–*Anser anser*, *Anser albifrons*, and *Anser fabalis–*are similar in length; the longest is that of *Anser anser* (16,738 bp) and the shortest is in Bean goose (16,688 bp). Compared with the other two species, some overlapping sequences are found in Bean goose mtDNA–in particular, in a 74-bp region between rRNA-Lus and tRNA^leu^. As in most Anseriformes, all genes are encoded on the same strand, and there are no missing or duplicated genes. Among the three mtDNAs, homologous regions comprise 16,239 bp, representing 97.12% of the entire genome. As seen in the three *Anser* genomes, these regions generally have the highest transition/transversion ratios and are more significant in closely-related species [14], [16]. All three *Anser* mitochondrial genomes are quite compact with very short intergenic regions (0.19% of the genome for *Anser anser*, 0.21% for *Anser albifrons*, and 0.32% for Bean goose).

### Comparison of Protein-coding Genes

When we compared the 13 Anseriform species with respect to predicted initiation and termination codons of 13 mitochondrial protein-coding genes (Table 3), we found that most protein-coding genes used ATG as start codons. Proteins in only a few species start with GTG, CTG, or ATA. Stop codons are also similar across species, with TAA, TAG, and T- occurring most frequently. Among the 13 protein-coding genes, specific examples include the following: the COI initiation codon is GTG and the termination codon is AGG in all 13 species; ND6 starts with ATG and ends with TAG, except in *Anser fabalis*, where it ends with TAA; ND1 starts with ATG and ends with AGG, except in *Anser albifrons*, where the stop codon is TAA; and ND2 starts with ATG and ends with TAG, except in *Anseranas semipalmata* and *Cygnus atratus*, for which the stop codon is TAA.

**Table 3:** Predicted initiation and termination codons for 13 mitochondrial protein-coding genes in 13 Anseriform species.

Gene	Predicted initiation and termination												
	A	B	C	D	E	F	G	H	I	J	K	L	M
ND1	ATG/TAA	ATG/AGG	ATG/AGG	ATG/AGG	ATG/AGG	ATG/AGG	ATA/AGG	ATG/AGG	ATG/AGG	ATG/AGG	ATG/AGG	ATG/AGG	ATG/AGG
ND2	ATG/TAG	ATG/TAG	ATG/TAG	ATG/TAA	ATG/TAG	ATG/TAG	ATG/TAG	ATG/TAG	ATG/TAG	ATG/TAA	ATG/TAA	ATG/TAA	ATG/TAA
COI	GTG/AGG	GTC/AGG	GTG/AGG	GTG/AGG	GTG/AGG	GTG/AGG	GTG/AGG	GTG/AGG	GTG/AGG	GTG/AGG	GTG/AGG	GTG/AGG	GTG/AGG
COII	GTG/TAA	GTG/TAA	CTG/TAA	GTG/TAA	GTG/TAA	GTG/TAA	GTG/TAA	GTG/TAA	GTG/TAA	ATG/TAA	GTG/TAA	GTG/TAA	GTG/TAA
ATP8	ATA/TAA	ATG/TAA	ATG/TAA	ATG/TAA	ATG/TAA	ATG/TAA	ATG/TAA	ATG/TAA	ATT/TGG	AT G/TAA	ATG/TAA	ATG/TAA	ATG/TAA
ATP6	ATG/TAA	ATG/TAA	ATG/TAA	ATG/TAA	ATG/TAA	ATG/TAA	ATG/TAA	ATG/TAA	ATG/TAA	ATG/TAA	ATG/TAA	ATG/TAA	ATG/TAA
COIII	ATG/TAA	ATG/T-	ATG/TAA	ATG/T-	ATG/T-	ATG/T-	ATG/T-	ATG/T-	ATG/T-	ATG/T-	ATG/TAA	ATG/TAA	ATG/T-
ND3	ATG/TAG	ATG/TAA	ATG/TAA	ATG/TAG	ATG/TAA	ATG/AGG	ATG/TAA	ATG/TAA	ATG/TAA	ATA/TAA	ATG/T-	ATG/TAA	ATG/TAA
ND4L	ATG/TAA	ATG/TAA	ATG/TAA	ATG/TAA	ATG/TAA	ATG/TAA	ATG/TAA	ATG/TAA	ATG/T-	GTG/TAA	ATG/TAA	ATG/TAA	ATG/TAA
ND4	ATG/T-	ATC/T-	ATG/T-	ATG/T-	ATG/T-	ATG/T-	ATG/T-	ATG/TAA	ATG/T-	ATG/T-	ATG/TAA	ATG/TAA	ATG/TAA
ND5	GTG/TAA	GTG/AGA	AGT/TAA	GTG/AGA	GTG/AGA	GTG/TAA	GTG/TAA	GTG/TAA	GTG/TAA	ATG/AGA	GTG/AGA	GTG/TAA	GTG/AGA
Cytb	ATG/TAA	ATG/TAA	AGT/TAA	ATG/TAA	ATG/TAA	ATG/TAA	ATG/TAA	ATG/TAA	ATG/TAA	ATG/TAA	ATG/TAA	ATG/TAA	ATG/TAA
ND6	ATG/TAG	ATG/TAG	ATG/TAG	ATG/TAG	ATG/TAG	ATG/TAG	ATG/TAG	ATG/TAG	ATG/TAG	ATG/TAG	ATG/TAG	ATG/TAG	ATG/TAA

Notes: A: *Anser albifrons* (NC_004539), B: *Anser anser* (NC_011196), C: *Branta canadensis* (NC_0071011), D: *Cygnus atratus* (NC_012843), E: *Cygnus columbianus* (NC_007691), F: *Mergus merganser* (NC_016723), G*: Cairina moschata* (NC_010965), H: *Anas platyrhynchos* (NC_009684), I: *Aythya americana* (NC_000877), J: *Anseranas semipalmata* (NC_005933), K: *Dendrocygna javanica* (NC_012844), L: *Anas formosa* (NC_015482), M: *Anas fabalis* (this study).

### Control Region Comparisons

**Figure 4:**
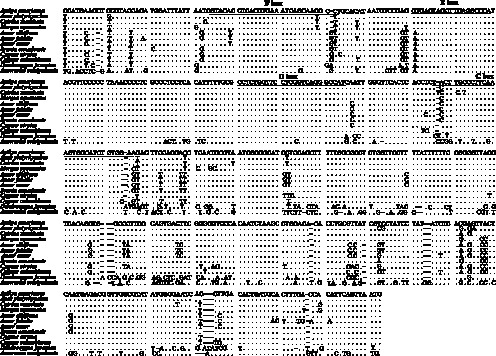
Structure of the central conserved domain in some Anseriform species. Dots indicate nucleotides with identity to the reference sequence, and dashes indicate indels.

The control region (D-Loop), responsible for transcription and replication, is the most variable mtDNA region, sometimes containing variable numbers and lengths of tandem repeats [22]. Three internal portions have been recognized in the control region: the 5′-peripheral domain, the central conserved domain, and the 3′-peripheral domain [36], [39], [45]; in Bean goose, these domains span positions 15,507–15,877, 15,878–16,335, and 16,336–16,688, respectively. Within the control region, the central conserved domain exhibits a rather homogeneous evolutionary rate among species [2], [18]. It contains conserved sequence blocks (CSB) B, D and F. In most avian mtDNA, C and E boxes are also found in this area [35], [40], [41]. The central domain is rich in C and T [16], [17]. In this region in Bean goose, C+T content (58.95%) is also higher than A+G content (41.05%). Complete sequences of Anseriform mitochondrial control regions have an average size of 1,100 bp, and range in size from 970 bp in the Maned goose (*Chenonetta jubata*) to 1,230 bp in the Crested screamer (*Chauna torquata*) [16]. Similar to other birds, there is only one control region, which is 1,182 bp long, in Bean goose mtDNA. Its nucleotide frequencies are not significantly different from other birds, and it does not contain any repeats. Comparative analyses of the structure and organization of control regions can help elucidate relationships in the Anseriformes [41]. Based on consensus alignment with central conserved domains of control region sequences of other Anseriform species, some conserved sequence boxes, such as F, E, D, and C, were identified (Fig. 4). These blocks are similar to those of other vertebrates [39], [42]. The average genetic distance (0.318) between control regions of *Anseranas semipalmata* and other studied species is higher than average (0.277). *Anseranas semipalmata* thus has the most divergent control region of the studied species (Fig. 4), suggesting that its D-loop sequence is evolving at a faster rate.

### Phylogenetic Analyses

Based on the results of our phylogenetic analyses, from which similar topologies were recovered, the inclusion of *Lophodytes cucullatus* and *Mergellus albellus* within the genus *Mergus* of tribe Mergini is supported, as suggested by an earlier study [32]. The genus *Somateria* is an early-diverging lineage in Mergini that is sometimes classified as a separate tribe, Somaterini [17], [43]. This view, supported by Donne-Goussé [16], is congruent with our results. Our phylogenetic trees suggest a close genetic relationship between Anatini and Aythyini, in accordance with the ideas of Gonzalez [10]. Within Anatidae–according to our study–Aythyini diverged earlier than Anatini and is monophyletic [10], [16]. Taxonomy and systematic relationships within Tadornini have been extensively debated [10], [30]. Tadornini monophyly is supported by phylogenetic analyses of control region data [10], but other molecular studies have shown the group to be non-monophyletic [16]. In our study, Tadornini contains the genera *Tadorna*, *Cairina*, and *Chloephaga*. *Cairina moschata* was formerly placed into the paraphyletic “perching duck” assemblage, but subsequently moved to the dabbling duck subfamily (Anatinae) [16], [30]. Analysis of Cyt b and ND2 mitochondrial gene sequences, however, indicate that this species might be more closely related to the genus *Aix* [16], [17], [44], [45], and suggest its placement in Cairinini rather than in Tadornini [16], [45]. Relationships among these taxa are not well-supported, however, and the addition of more complete mitochondrial genome sequence data is needed. In our analyses, the Bean goose, a member of tribe Anserini, has a close genetic relationship with fellow tribal members *Anser albifrons*, *Anser anser*, and *Anser cygnoides.* Anserini and Cygnini are shown to be sister groups, together composing the Anserinae. In our study, their placement is similar to that in phylogenetic trees generated from control region gene data [16].

The classification status of *Dendrocygna* and the Anseranatidae has been a subject of debate. Some authors have placed *Dendrocygna* inside the subfamily Anserinae and included Anseranatidae in Anatidae [17], while other authors consider them to be two different lineages [46]. Our molecular results, both those based on complete mitochondrial genome data and those derived from combined sequences of Cyt b, ND2, and COI, suggest that *Dendrocygna* does not belong to Anserinae, but represents an independent subfamily. The situation is similar for *Anseranas semipalmata*, which appears to be an independent family. Phylogenetic analyses based on complete mtDNA or concatenated genes (ND2+Cytb+COI) both clearly strongly support *Dendrocygna* (*Dendrocygna javanica*) as an independent subfamily, Dendrocygninae, within the family Anatidae, as well as the existence of Anseranatidae (*Anseranas semipalmata*) as an independent family [30].

### Comparison of Phylogenies Based on Complete mtDNA with those Derived from Combined ND2, Cyt b, and COI Data

Mitochondrial DNA has been extensively used as a molecular marker for resolving phylogenetic relationships at different taxonomic levels in difficult animal groups [47], [48]. Results are sometimes very controversial, however, because they are derived from a small set of specific gene markers [10], [16], [17]. Mitochondrial DNA–either single genomes or a combination of markers–has been used to resolve phylogenetic relationships among Anseriform species, but the results are still considered controversial [10], [16], [36]. Several recent analyses have demonstrated that phylogenies based on complete mtDNA are better supported than those based on individual genes or partial mitochondrial genomes [1], [10], [23]. Complete mtDNA data may thus provide new insights into Anseriform higher-level systematics. Currently, however, complete mtDNA genomes of only 13 Anseriform species, including *Anser fabalis* from our study, have been sequenced and deposited in GenBank, limiting the utility of Anseriform mitochondrial genomes as molecular markers for resolving phylogenetic relationships. In our study, tribal-level phylogenetic relationships in Anseriformes based on complete mtDNA are similar to those derived from combined ND2, Cyt b, and COI gene sequence data, with only some minor topological differences due to the limited taxon sampling in the case of complete mtDNA (Figs. 2 and 3). We therefore conclude that in the absence of enough completely sequenced mitochondrial genomes, the use of combined ND2, Cyt b, and COI sequence data is a workable solution for reconstructing phylogenetic relationships among Anseriform species.

## Materials and Methods

### Specimen Collection

A Bean goose (*Anser fabalis*) injured by power lines was found in the wild in the Shengjin Lake National Nature Reserve, Anhui, China in October 2010. The bird was treated for its injuries but later died (The Shengjin Lake National Nature Reserve is authorized to administer medical treatment to animals by the Anhui Provincial Nature and Wildlife Conservation Station, a provincial government agency for wildlife in Anhui Province). Tissues from the dead bird were field-stored at −20°C at the Institute of Biodiversity and Wetland Ecology, Anhui University. Permission to collect the bird in the wild and use the tissue was issued by the Shengjin Lake National Nature Reserve.

### DNA Extraction, PCR Amplification, and Sequencing

Whole genomic DNA of Bean goose was isolated from muscle tissue using the phenol/chloroform method. Extracted DNA was examined on a 1.0% agarose/TBE gel and stored at −20°C as a template for PCR.

Based on alignment of complete mtDNA sequences of *Cygnus atratus* (NC_012843), *Anas platyrhynchos* (NC_009684), and *Aythya americana* (NC_000877), we designed three primer pairs (primer sets 9, 11, and 14) using Primer 5.0. We also employed other primers developed for use with *Anas platyrhynchos* (Jianchang duck) [33]. These primers were used to amplify and sequence the complete mitochondrial genome of Bean goose (Table 4). All generated sequences were less than 1200 bp each, with each segment overlapping the next by 80–100 bp.

**Table 4:** Primer sequences used in this study.

Primer No	Amplified region	Forward primer sequence (5′→3′)	Reverse primer sequence (5′→3′)
1	840–2106	CCACTACCCGAGACCTACG	TAAGTCTTTTGTCCGCAGGCAT
2	1997–3190	ATAGGGCTATTTAGTGAATGCT	TGAGTATTCTAAGTACACCTTC
3	3017–5121	GTCACCCTCCTCATAAGCCA	GTTGCTGGAGATTGTGATTGT
4	4944–7091	TTACCAAAAACATAGCCTTCAG	GTTGCTGGAGATTGTGATTGT
5	6886–8340	GATCAAAACTCTCCATACTTCC	TCGGTCGGTTAGTAGCATTGT
6	8210–9346	TGGCTATCTTCTCACTTCACCT	GGGTAGGATTGTTCAGATTAGT
7	9176–10285	ACCACGCTCTGATTGTTGCCT	TTGTGTGGTGGGGTGAATGT
8	10127–11367	CGACTTTCCACCATCCAACT	CTTCGTGGTATTCTATTGCCT
9	11017–12122	TACTAAACACAGCAATCCTCCT	GAATTTTGGTGGGGACAGTAG
10	11944–12925	CTACACCTGAGCTTCTACT	GAGACAGATTGAGCTAGT
11	12747–13834	TCTGACTACCAAAAGCCCAC	GTGAGTAGTGTGAGGGAGTT
12	13685–14810	GAGTTAAATCAACAAGAGCT	GTCCGATGGCTACTATTAT
13	14702–15767	ACTAGCCACCAACCAAACAG	CGAAGTTTCATCAGGCAGAG
14	15063–93	ATGATCTTAACCACACAGACC	GTTGTCCGATGATGATGAATG
15	16547–1128	CCTMCTRCTCACTCTTAT	CTATGCACGATATGCAT

Note: M refers to A and C, R refers to A and G.

PCR amplifications were carried out in 25-µl volumes containing 100 ng template DNA, 2.5 µl of 10× reaction Buffer, 1 µl of 25 mM MgCl_2_, 2 µl of 2 mM dNTPs, 1 µl of each 10 mM primer, 0.5 U Taq DNA polymerase (Trans Taq-T DNA Polymerase, Beijing, China), and sterile doubly-distilled water to final volume. PCR amplification conditions were as follows: denaturation for 5 min at 94°C, followed by 30 cycles of denaturation for 30 s at 94°C, annealing for 30 s at 49–55°C (depending on primer combinations), and elongation for 2 min at 72°C, and a final extension step for 10 min at 72°C. PCR products were purified using a V-gen PCR clean-up purification kit, and were then bidirectionally sequenced by Sangon Biotech (Shanghai, China).

### Sequence Analysis

Sequences were checked and assembled using the programs Seqman (DNASTAR 2001), BioEdit, and Chromas 2.22, and then adjusted manually. Protein-coding genes were identified by comparison with known complete mtDNA sequences of Anseriform birds using Sequin 11.0. The 22 tRNA genes were identified using the software package tRNA Scan-SE 1.21 (http://lowelab.ucsc.edu/tRNAscan-SE), with their cloverleaf secondary structures and anticodon sequences determined using DNASIS (Ver.2.5, Hitachi Software Engineering). Two rRNAs were identified by comparison with complete mtDNA sequences of other Anseriformes available in GenBank. The complete mitochondrial genome sequence of Bean goose has been deposited in GenBank under accession number NC_016922.

### Phylogenetic Analyses

To study the phylogeny of Anseriformes, phylogenetic trees were reconstructed using ML and BI methods, with *Gallus gallus* (NC_001323) used as an outgroup. Two sets of phylogenetic trees were generated, one based on complete mtDNA for 13 Anseriform species, and the other derived from multiple sequence alignments of several mitochondrial gene regions for 36 typical Anseriform species (Table 5). Avian species generally exhibit moderate levels of sequence divergence in mitochondrial gene regions, especially in protein-coding genes such as Cyt b, ND2, and COI; a combination of these three genes is suitable for resolving phylogenetic problems at different taxonomic levels, ranging from related species to genera and families [14], [15]. Because we were interested in resolving relationships at these taxonomic levels within Anseriformes, we chose Cyt b, ND2, and COI sequence data for use in the second phylogenetic analysis.

**Table 5:** GenBank accession numbers for the 36 Anseriform species in this study.

Species	Accession number (Cyt b, ND2, COI)	Species	Accession number (Cyt b, ND2, COI)
*Anseranas semipalmata*	NC_005933,NC_005933,NC_005933	*Somateria spectabilis*	EU585662,EU585725, JN801375
*Dendrocygna javanica*	NC_012844,NC_012844,NC_012844	*Melanitta perspicillata*	EU585652, EU585715, DQ434654
*Cygnus atratus*	NC_012843,NC_012843,NC_012843	*Melanitta nigra*	AF515263, AF515267, DQ434651
*Anser albifrons*	NC_004539,NC_004539,NC_004539	*Bucephala islandica*	EU585635, EU585698, DQ434501
*Anser fabalis*	This study	*Bucephala clangula*	EU585634, EU585697, DQ434493
*Chloephaga picta*	AF515262,AF515266, FJ027352	*Bucephala albeola*	EU585633, EU585696, DQ434491
*Mergus squamatus*	NC_016723,NC_016723,NC_016723	*Lophodytes cucullatus*	EU585650, EU585713, DQ434630
*Mergellus albellus*	EU585653, EU585716, JN801325	*Mergus merganser*	EU585654, EU585717, DQ434672
*Cairina moschata*	NC_010965,NC_010965,NC_010965	*Mergus serrator*	EU585654, EU585718, DQ434676
*Tadorna tadorna*	AF059113,AF059173,GU572125	*Anser anser*	NC_011196,NC_011196,NC_011196
*Tadorna ferruginea*	EU585664,EU585727,GQ482749	*Branta canadensis*	NC_007011,NC_007011,NC_007011
*Anas formosa*	NC_015482,NC_015482,NC_015482	*Netta rufina*	GU571988, EU585657, EU585720
*Anas clypeata*	AF059062,AF059174,DQ434273	*Aythya americana*	NC_000877,NC_000877,NC_000877
*Anas sibilatrix*	AF059108,AF059168,FJ027108	*Aythya ferina*	NC_000877, NC_000877, NC_000877
*Anas strepera*	AF059109,AF059169,DQ434300	*Cygnus columbianus*	NC_007691,NC_007691,NC_007691
*Anas bahamensis*	AF059058,AF059119,JQ174013	*Cygnus olor*	HM063581, HM063568, GU571856
*Anas acuta*	AF059055,AF059116,DQ434263	*Anas crecca*	AF059064, AF059124, JN703195
*Somateria mollissima*	AF515264,AF515268,DQ434750	*Anas platyrhynchos*	NC_009684,NC_009684,NC_009684

Before reconstructing phylogenetic trees, sequence alignment was carried out using the 13 complete mtDNA Anseriform genomes and the three concatenated data sets for 36 Anseriform species using ClustalX 1.8, followed by manual adjustment. ML analyses were performed in PAUP (version 4.0b8) using TBR branch swapping (10 random addition sequences) and a general time-reversible model with invariant sites and among-site variation (GTR+I+Γ); this model was selected as the best fit model of evolution using Modeltest (version 3.06) based on the AIC criterion. Support for internal branches in the ML tree was evaluated via the bootstrap test with 100 iterations. Bayesian inference of phylogeny was performed using the program MrBayes 3.1.2, with the same best fit substitution model used as the one selected for the ML analysis. MrBayes 3.1.2 simultaneously initiates two Markov Chain Monte Carlo (MCMC) runs to provide additional confirmation of convergence of posterior probability distributions. Analyses were run for one million generations until the average standard deviation of split frequencies was less than 0.01, which indicated that convergence was reached. Chains were sampled every 1000 generations.
