# Correction: The Complete Mitochondrial Genome of Bean Goose (*Anser fabalis*) and Implications for Anseriformes Taxonomy

**DOI:** 10.1371/annotation/dcc97c7a-057d-4331-96e6-8e8898c6bf64

**Published:** 2013-08-05

**Authors:** Gang Liu, Lizhi Zhou, Lili Zhang, Zijun Luo, Wenbin Xu

Figures 2 and 4 are corrupted in the HTML article. Please use the following links to access the proper versions of these figures.

Figure 2: 

**Figure:**
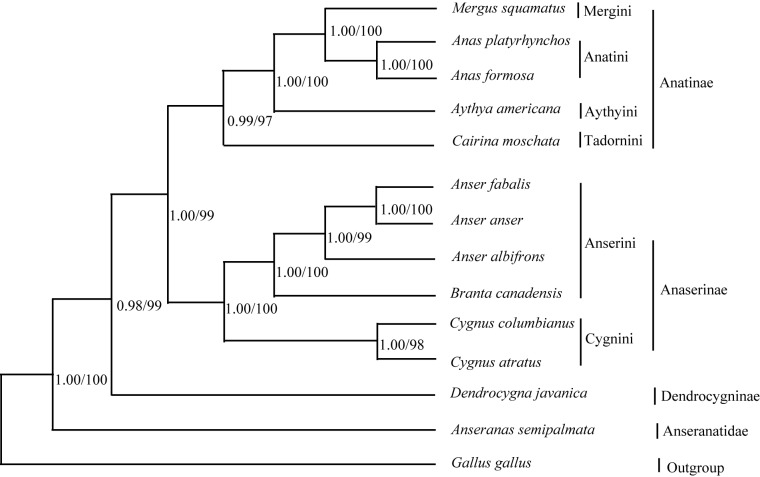


Figure 4: 

**Figure:**
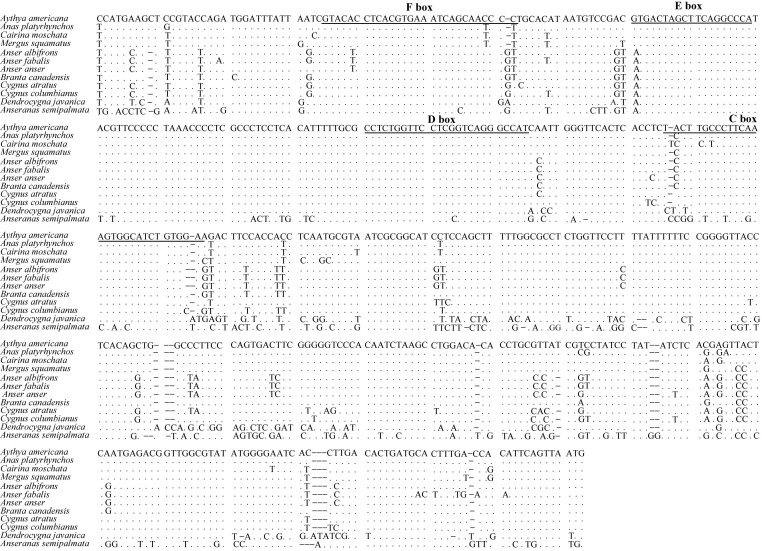


Figure 3 incorrectly refers to "Anas clypeata" in the diagram. This has been replaced with "Anas sibilatrix". The correct figure can be seen at the following link:

http://plosone.org/corrections/pone.0063334.g003.tif

